# Improved Non-Invasive Diagnosis of Bladder Cancer with an Electronic Nose: A Large Pilot Study

**DOI:** 10.3390/jcm10214984

**Published:** 2021-10-27

**Authors:** PierFrancesco Bassi, Luca Di Gianfrancesco, Luigi Salmaso, Mauro Ragonese, Giuseppe Palermo, Emilio Sacco, Rosa Arboretti Giancristofaro, Riccardo Ceccato, Marco Racioppi

**Affiliations:** 1 Department of Urology, Fondazione Policlinico Universitario “Agostino Gemelli” IRCCS di Roma, Università Cattolica del Sacro Cuore di Roma, Largo Agostino Gemelli, 8, 00168 Rome, Italy; pierfrancesco.bassi@policlinicogemelli.it (P.B.); mauro.ragonese@guest.policlinicogemelli.it (M.R.); giuseppe.palermo@policlinicogemelli.it (G.P.); emilio.sacco@policlinicogemelli.it (E.S.); marco.racioppi@policlinicogemelli.it (M.R.); 2 Department of Management and Engineering, Università di Padova, 35122 Padova, Italy; luigisalmaso.unipd@gmail.com (L.S.); rosa.arboretti@unipd.it (R.A.G.); riccardo.ceccato91@gmail.com (R.C.)

**Keywords:** electronic nose, non-invasive diagnosis, volatile organic compounds, bladder cancer

## Abstract

Background: Bladder cancer (BCa) emits specific volatile organic compounds (VOCs) in the urine headspace that can be detected by an electronic nose. The diagnostic performance of an electronic nose in detecting BCa was investigated in a pilot study. Methods: A prospective, single-center, controlled, non-randomized, phase 2 study was carried out on 198 consecutive subjects (102 with proven BCa, 96 controls). Urine samples were evaluated with an electronic nose provided with 32 volatile gas analyzer sensors. The tests were repeated at least two times per sample. Accuracy, sensitivity, specificity, and variability were evaluated using mainly the non-parametric combination method, permutation tests, and discriminant analysis classification. Results: Statistically significant differences between BCa patients and controls were reported by 28 (87.5%) of the 32 sensors. The overall discriminatory power, sensitivity, and specificity were 78.8%, 74.1%, and 76%, respectively; 13/96 (13.5%) controls and 29/102 (28.4%) BCa patients were misclassified as false positive and false negative, respectively. Where the most efficient sensors were selected, the sensitivity and specificity increased up to 91.1% (72.5–100) and 89.1% (81–95.8), respectively. None of the tumor characteristics represented independent predictors of device responsiveness. Conclusions: The electronic nose might represent a potentially reliable, quick, accurate, and cost-effective tool for non-invasive BCa diagnosis.

## 1. Introduction

To date, cystoscopy remains the gold standard in bladder cancer (BCa) diagnosis, but it is invasive and uncomfortable. Urinary cytology and radiology play an important role in follow-up but have some limitations in the diagnostic setting and still remain exam operator sensitive [1,2,3,4]. Hence, many efforts are focusing on the development of accurate non-invasive diagnostic tests for BCa.

Volatile organic compounds (VOCs) represent natural markers of pathophysiological mechanisms in the human body [5]. They are normally generated by biochemical processes, including oxidative stress and lipid metabolism, or can be absorbed from the external world through ingestion, inhalation, or skin contact [6]. An electronic nose is an electronic sensing device intended to detect odors or flavors. The term “electronic sensing” refers to the ability to reproduce human senses using sensor arrays and pattern recognition systems. Once the VOCs are bounded to the detector, it is possible to obtain and analyze the associated electronic signal with specific software tools.

BCa is associated with the presence of specific VOCs in gas emitted from urine samples, due to the fact that during tumor growth, protein changes in malignant cells lead to peroxidation of cell membrane components, producing VOCs [7,8]. Therefore, VOCs in the urine headspace (the gas or empty space above the contents of a sealed container) from BCa patients might play a role as a diagnostic tool. These compounds have been revealed by pilot studies on the use of the olfactory capacity of dogs [9,10] and the use of mass spectrometry [11]. They were detected even with an electronic nose with sensitivity and specificity rates of up to 70% for BCa [12], thereby opening new perspectives in this field.

In this pilot study, we evaluated the potentially reliable and feasible diagnostic performances of a VOC’s sensor device in BCa diagnosis, validated by updated statistical methods, intended as validated and current methods of managing data and results. The hypothesis was to evaluate the ability of the electronic nose to detect BCa by analyzing the VOCs; in this setting (pilot study), any stage of BCa patients was investigated.

## 2. Materials and Methods

### 2.1. Patients’ Characteristics

**Figure 1:**
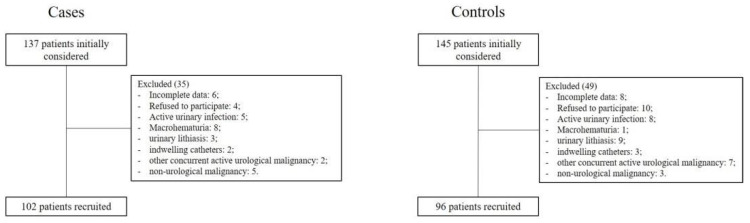
Flowchart.

A prospective, single-center, controlled, non-randomized phase 2 study was carried out. A total of 198 consecutive subjects were recruited: 102 patients with proven BCa and 96 controls (without BCa) (Figure 1).

The patients with confirmed BCa were enrolled before undergoing trans-urethral resection of bladder tumor (TURB-T). The controls were enrolled in the outpatient setting, from among patients in follow-up for non-complicated benign prostatic hyperplasia or urinary incontinence or for urinary lithiasis with long disease-free status. The controls did not report macrohematuria or urinary symptoms consistent with a bladder lesion. They underwent imaging of the urinary tract, with negative evidence of Bca. Neither urinary cytology nor cystoscopy evaluations were done, since no suspicions of BCa were detected. Subjects with proven active urinary infections, urinary lithiasis, indwelling catheters, and other active concurrent urological and non-urological malignancies were excluded.

In the case of misclassification by the electronic nose as false-positive, and after obtaining informed consent, the controls underwent serial assessments with further urinary cytology and radiological evaluations of the urinary tract in order to rule out BCa. We performed radiological imaging of the urinary tract (ultrasound and UroCT or UroMR in the event of a suspicious or doubtful finding at the first-level exam) according to EAU guideline recommendations: “Use renal and bladder ultrasound and/or computed tomography-intravenous urography (CT-IVU) during the initial workup in patients with hematuria” (strength rating: strong) [1]. We performed voided and washing cytology in order to complete a hypothetical initial workup for suspected bladder cancer. None of the false-positive controls required an endoscopic evaluation, and none of them received a bladder cancer diagnosis.

We did not perform a prior sample size calculation but based the sample size on the recommendations of Teare et al. [9]: “If the primary outcome is binary, a total of at least 120 subjects (60 in each group) are required in the pilot trial”. We therefore enrolled at least 60 subjects per group. We joined the STARD guidelines for reporting diagnostic studies (flowchart).

### 2.2. Sample Management

Urine headspace measurements were performed using the Cyranose 320^®^ device (Sensigent), a volatile gas analyzer equipped with 32 sensors. The urine headspace is the gas or empty space above the contents of a sealed container.

We collected 10–25 mL of urine. Urine samples were taken in the morning after the evaluation of inclusion and exclusion criteria and acquisition of signed informed consent. Samples were collected in the morning since the urine is generally more concentrated (due to the length of time, the urine is allowed to remain in the bladder) and, therefore, contains relatively higher levels of cellular elements and analytes that could emit a higher level of VOCs. The urine samples from BCa patients were collected before TURBT and from controls in outpatient settings. Sterile urine samples were collected after at least 12 h of fasting from solids (in order to minimize possible carcinogens that are digested, e.g., nitrosamines in meat products) and liquids. The purposes of using morning void urine were standardization of the sample collection (such as in its timing) and the attempt to empirically minimize the potential lack of activation of the sensors due to less concentrated urine (the inability to identify individual molecules that compose the odor might be overcome by a “richer” headspace); the latest aspect surely deserves to be further explored. The urine samples were then immediately placed in a sealed container at 37–38.5 °C for stabilization (at least for 1 h to obtain a steady headspace). The stabilized samples were analyzed within 2 h of collection after device calibration. The calibration consisted of 10 s analysis of the headspace of a container with 10 mL of sterile saline (the container had the same characteristics as the ones used for the evaluation of the headspace of urine in the exam). The collection, stabilization, and analysis were standardized to avoid bias regarding these steps.

This tool has not yet been independently validated; one of the purposes of the pilot study was to pave the way for the standardization of the proposed system in order to precisely validate it independently.

### 2.3. Electronic Nose

Electronic noses include three major parts: a sample delivery system, a detection system, and a computing system. Essentially the instrument consists of headspace sampling, a chemical sensor array, and pattern recognition modules to generate signal patterns that are used for characterizing odors [13]. Cyranose 320^®^ has a matrix of 32 sensors of carbon black polymer. When an odor (chemical input) is presented to the electronic nose, it causes a physical change in the sensors, which is detected by the transducers and converted into an electrical signal, creating a specific signature or smellprint. The readings of the 32 sensors were taken and recorded in the software. All readings of the 32 sensors were extracted and further analyzed. Each sensor is not specific for a singular and different VOC; indeed, each different VOC stimulates the activation of one or more than one sensor. The 32 sensors constitute a matrix of carbon black polymers; the use of different types allows avoiding or at least minimizing the disadvantages presented by each one separately and maximizing their advantages. The 32 sensors are not identical, and each sensor provides a response related to the variation in a physical quantity that characterizes the sensor itself, such as the variation in conductivity, the resonance frequency, or the mass. Like the human nose, the electronic nose does not perform a chemical speciation of the analyzed odor, so it is not able to identify the individual molecules that compose it, but the set of sensors produces a sort of “olfactory imprint,” which can be classified on the basis of a reference database acquired by the instrument in a preliminary training phase. On the basis of this concept, the electronic nose, in this setting and specific (exploratory) phase of study/evaluation, is not able to distinguish a single VOC from a cluster.

### 2.4. Results Management and Statistics

The analyses were repeated at least 2 times per sample to validate the VOCs’ nature by controlling the Euclidean distance between the values. We considered all measurements in the analyses. The results obtained with the electronic nose were directly compared to the final histopathological reports after TURB-T or RC (cytology was performed only in the case of recurrent HG tumor or in the case of suspected Cis).

A non-parametric combination (NPC) of the dependent permutation test methodology [14,15] was performed on a total of 198 sample subjects in order to predict whether the subject belonged or did not belong to the BCa patients’ group. We computed partial permutation tests for the two independent samples on subsamples determined by one of the recorded characteristics of the patients (i.e., gender, smoking, and comorbidity), evaluating the ability of each sensor in discerning people from the two different health status groups. We then provided *p*-values adjusted for multiplicity using a closed testing procedure, controlling the FWE rate. The NPC let us consider a test as positive or negative in terms of detection. We applied the Tippet combination function [16] to combine the partial tests obtained from the analysis of subsamples determined by levels of the same factor (such as male/female or smoker/non-smoker/former smoker). This allowed us to evaluate the global performances of each sensor.

We performed permutation tests on BCa patients to test the sensors’ ability to detect the potential effect of different tumor types or comorbidities on the VOCs’ composition in the urine headspace. The cutoff for sensor activation was generated by the nonparametric statistics, revealing the activation of the sensor by the presence of VOCs but not the concentration of VOCs. We performed a discriminant analysis classification of multiple repeated detections to classify observations into groups and to describe the relative importance of variables for distinguishing between groups. This analysis allowed for the identification of the level of sensor activation: taking into account all measurements, all sensors activated in each measurement in the BCa group were considered as the most effective. We assumed misclassification as a false-negative or a false-positive result.

Categorical variables were evaluated as counts and percentages, and x^2^-distribution was used; continuous variables were evaluated by using mean ± standard deviation; parametric and nonparametric variables were evaluated with the *t*-test and the chi-square test; logistic regression was performed; and Pearson’s test was used for correlation analysis. Statistical significance was set at *p* < 0.05.

## 3. Results

We reported baseline and tumor characteristics in Table 1 and Table 2, respectively. Considering baseline characteristics, there was no statistically significant difference between the two groups.

The reached sensitivity to detect BCa by using the electronic nose was 74.1% (10.8–100%) and the specificity 76% (3–95.8%) (AUC 0.563 (0.392–0.752)) when considering the analysis of all sensors. When we considered sensors for which the null hypothesis of equality was rejected (by selecting the most efficient sensors, which amounted to 9 of the 32), the sensitivity increased to 91.1% (72.5–100) and the specificity to 89.1% (81–95.8) (AUC 0.601 (0.578–0.626)).

The overall discriminating power rate by the discriminant analysis classification was 78.8%; consequently, 21.2% of all subjects (42/198) were not correctly classified (or misclassified): 13/96 (13.5%) controls classified as false positive and 29/102 (28.4%) BCa patients as false negative. In the N-MIBC group, the rate of false negatives (misclassification rate) was 22.2% in the case of low-grade pTa, 11.1% for high-grade pTa, 18.2% for high-grade pT1, 6.1% for low-grade pT1, and 1.6% for solitary/concomitant pCis. In the MIBC group, the rate of false negatives (misclassification rate) was 23.8%. The misclassified tumors had a mean size of 2.69 ± 1.08 cm (range 1.5–5): 2 cm (range 1–4) for low-grade N-MIBC patients, 3.2 cm (range 1–6) for high-grade N-MIBC patients, and 3.4 ± 1.14 cm in MIBC patients. Other investigations (cystoscopy, cytology, and/or radiology) succeeded in diagnosing cancer in all these patients.

By comparing the mean values detected by each sensor, the difference was not statistically significant (*p* = 0.20) in the comparison between BCa patients and controls. By comparing the absolute values detected by each sensor, 18 of 32 sensors revealed statistically significant differences between the two groups (they were defined as the most responsive).

Age, history of acute myocardial infarction (AMI), presence of BCa, and the mean sensor activation value represented the variables determining the positivity (i.e., activation level) of sensors (*p* < 0.05) in the multivariate analysis of all subjects corrected for the sensors’ response. In the multivariate analysis of BCa patients, none of the tumor characteristics (histology, grade, stage, focality, mean size, first diagnosis, median time from first diagnosis) emerged as an independent predictor of the sensors’ response.

Interestingly, we reported a linear correlation between the level of the sensors’ activation and the presence of BCa (r = 0.08, *p* = 0.01).

**Figure 2:**
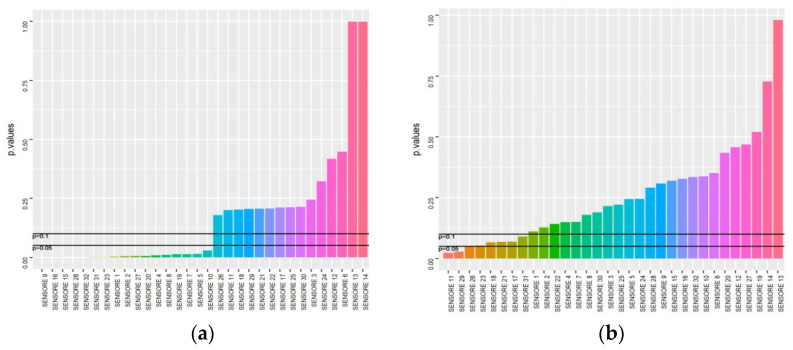

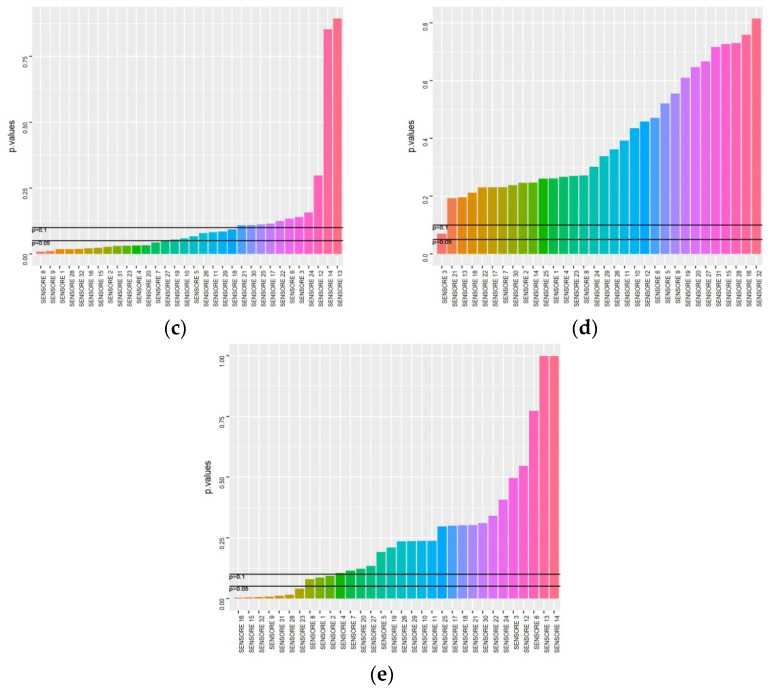
Measurement patterns identified (tumor characteristics): (**a**) NMIBC vs. MIBC; (**b**) high vs. low grade; (**c**) urothelial vs. other; (**d**) monofocal vs. multifocal; and (**e**) small vs. large.

Moreover, by considering all subjects, we reported statistically significant differences in VOC measurement-identified patterns between controls and BCa patients and regarding gender (for 28/32 sensors), smoking status (for 30/32 sensors), and some comorbidities (for up to 27/32 sensors, such as in the case of diabetes mellitus and COPD) (Appendix A). Furthermore, we reported statistically significant differences (mean *p* < 0.05) in the discriminating power rate in BCa patients by comparing N-MIBC to MIBC for 17/32 (53.1%) sensors (Figure 2a); high- to low-grade tumors for 8/32 (25%) sensors (Figure 2b); pure urothelial to other histopathological differentiation for 14/32 (43.8%) sensors (Figure 2c); and small to large tumors for 7/32 (21.9%) sensors (Figure 2e).

## 4. Discussion

The electronic nose is extensively used in several type of malignancies, such as respiratory diseases (including lung cancer), colorectal cancer, and prostate cancer [17]. The research on VOCs was initially developed by using the olfactory capacity of dogs; Willis et al. demonstrated the presence of specific BCa VOCs with the aid of trained dogs, achieving interesting sensitivity and specificity rates up to 86% and 92%, respectively [10]. Successively, with gas chromatography (GC) and mass spectrometry (MS), the electronic nose has been introduced in the field with the potential to discriminate between urine samples of BCa patients and controls.

Undoubtedly, gas sensor arrays offer practical advantages in VOCs’ detection, but they are not able to identify the chemical nature of VOCs [18]. Despite this limit, a GC device combined with a VOC recognition pattern achieved accuracy rates of 93–100% in a pilot study on BCa [19]. Gas chromatography with mass spectrometry (GC-MS) is actually recognized as an important analytical technique in the field of metabolomics due to high sensitivity, reproducibility, and peak resolution [20]. Jobu et al. showed that the score plots differed between BCa patients and controls on principal components analysis (PCA) mapping. The authors identified five substances as BCa biomarkers but only with a potential role: ethylbenzene, nonanoyl chloride, dodecanal, (*Z*)-2-nonenal, and 5-dimethyl-3(2*H*)-isoxazolone. Interestingly, the authors reported that medication could only slightly influence urine odor. The authors concluded that a urine-odor-based BCa diagnosis might prove more sensitive than urinary cytology [21].

In the study by Khalid et al., by using GC-MS, the two-group linear discriminant analysis (LDA) correctly identified 24/24 (100%) cancer cases and 70/74 (94.6%) controls. For partial least-squares discriminant analysis (PLS-DA), the correct leave-one-out cross-validation (LOOCV) prediction values were 95.8% (23/24 cancer cases) and 94.6% (69/74 controls) [19].

It is critical to note that in the case of more advanced tumors (such as muscle invasive), patients might be misclassified as negative regardless of the method applied in VOCs’ detection. This is possibly due to tumor by-products overwhelming the VOCs. Previous canine olfactory studies support this hypothesis: high-grade advanced cancers were missed more frequently than low-grade ones [10]. Surely, the electronic nose is not intended as a diagnostic tool for advanced disease, which deserve to be comprehensively detected and studied by standardized and consolidated tests.

An electronic nose is “an instrument which comprises an array of electronic chemical sensors with partial specificity and an appropriate patter-recognition system, capable of recognizing simple or complex odours” [22]. This device mimics the mammalian olfactory system and can identify different complex odors, comparing the incoming odor with patterns previously learned [23]. When an odor (chemical input) is presented to the electronic nose, it causes a physical change in the sensors, which is detected by the transducers and converted into an electrical signal, creating a specific signature or smellprint [22]. The rise and decline in the signal depend on some parameters: nature of the odor (type and concentration of the compounds), reaction and diffusion between odor and sensors, type of sensor, and ambient conditions [22].

Methods based on mass spectrometry analysis can detect and identify which compounds present in air samples are useful for pathophysiologic research [24]. Yet, these methods are time consuming and expensive and depend on a skilled operator; this makes them difficult to be used in real clinical settings. Electronic noses have the potential to overcome these disadvantages because they are relatively inexpensive and easy to use and provide rapid analysis [22]. To achieve this goal, it is necessary to create a prediction model with a training set of samples and external validation of the model for further application. Few studies have examined the ability of electronic nose technology to assess the VOCs’ role in BCa diagnosis. Bernabei et al. confirmed a close correlation between VOCs in the urine headspace and urological cancers by processing data with both PCA and PLS-DA [25]. The latest experience with an electronic nose was reported by Heers et al. calculating the Mahalanobis distance and LDA. After storage at −20 °C, the system correctly detected 28/30 BCa samples and 26/30 controls (*p* < 0.01), achieving sensitivity and specificity rates of 93.3% and 86.7%, respectively. Similar results were obtained after storage at −80 °C (sensitivity and specificity both 93.3%). However, the authors stressed the need for further research to test for possible confounders [26]. One of the differences with the research of Heers et al. was the different test methodology. Moreover, the authors stabilized the specimen at −20 °C and −80 °C, and this surely added costs to the entire process. Our research did not include the lowering of the sample temperature, and we stabilized the collected specimen in a sealed container at 37–38.5 °C; anyway, this surely represents another point of discussion and further improvement of the technique. Furthermore, unlike in the study of Heers et al., our study cohort was larger and more complex (different cases at different stages), and the patients were consecutive.

Matsumoto et al. explored the electronic nose ability to distinguish urological diseases by the urinary odor feature: they compared 36 untreated patients with BCa, 29 with urolithiasis, 10 with urinary tract infection, and 27 healthy volunteers. The authors used a device with only two sensors, and they established the quantity of odor with the value of θ detected during a measurement. They reported that the angle of the two sensors (θ) depended on the kinds of chemical substances, thus defining θ as the feature of odor. The resulting diagnostic sensitivity for bladder cancer was 61.4%, and specificity was 52.8%: the authors concluded that this non-invasive instrument is useful for distinguishing bladder cancer from other benign conditions [27].

The device showed high discriminatory power, with promising accuracy, sensitivity, and specificity rates. However, our data was not fully comparable with other experiences (Table 3) because of the differences in sampling and analytical methods. The lower rates achieved in our study could be related to the larger and more varied sample of patients.

In this pilot study, we excluded BCa patients with hematuria or bacteriuria to minimize possible bias related to conditions assumed as confounders: inflammatory cells and/or red blood cells might affect (even if with controversial results) the detection accuracy of the electronic nose [25]. After selecting NMIBC patients and controls, the overall discriminatory power was 81.2%. This meant that 11.6% of controls were misclassified as cases of cancer and 37.3% of cancer patients were overlooked. The highest misclassification rate (22.2%) was reported in low-grade pTa patients, the least aggressive NMIBC (which does not warrant such intensive surveillance as high-risk disease). In the case of false-positive results, we decided not to perform cystoscopy in controls due to the exploratory nature of this pilot study, whose results, even if promising, could not justify an uncomfortable, costly, and not-free-from-complications procedure in patients without suspicions of bladder cancer.

The misclassification rate surely deserves to be fully explored, as urologists might not be comfortable with these data. However, we believe that selecting the most responsive sensors might overcome this limitation. The variations in the misclassification rates could be justified by many events potentially occurring in any phase of the management of samples or results. One of the aims of this study was precisely to lay the groundwork to standardize the entire procedure in order to minimize variations in the management and then in the results. Effectively, some sensors revealed a sensitivity near 100% and a specificity of up to 95.8%, assuming that some cancers emit specific VOCs.

Data from the multivariate analysis might be further explained by the characteristic heterogeneity of BCa and BCa patients, difficult to unequivocally classify. The overall detection ability of the electronic nose was not influenced by smoking habits, despite the fact that in the subsequent analysis, we reported that an active smoker status affects the urinary headspace composition. Moreover, 27 sensors (84.4%) were able to distinguish subjects with or without comorbidities; other VOC studies have provided no data on the issue of comorbidities; therefore, our findings could surely represent a basis for future research.

The Cyranose 320^®^ device could logistically be widely available at some point, considering cost (that could be quickly amortized in the light of the high number of patients who could avoid more costly tests), size (4 × 8.8 × 2 in (10 × 22 × 5 cm^3^), 30 oz (0.9 kg)), upkeep (1 year warranty), ease of use (with a short learning curve), and transportability.

Several urine-based biomarkers (Table 4) have been previously validated and partially accepted for clinical use, since current commercially available urinary biomarker-based tests are not sufficiently validated to be widely used in clinical practice yet. These tests were mainly compared to the diagnostic accuracy of the standard urinary cytology, and most of the proposed molecular markers were able to improve the sensitivity with similar or lower specificity when compared to urinary cytology. However, the variability in results among the different studies was strong [28,29].

The main limitations of our study were small sample sizes, strict inclusion/exclusion criteria, heterogeneity between patients and controls, unbalanced misclassification rates. the differences in sensor reading seeming patient specific; a lack of comparison with cytology, the inability of the electronic nose to identify a specific VOC, the lack of further analysis of patients’ subcategories, and the conception of the study on the general performance of the electronic nose and not on the best sensors. Another significant limitation of the study was represented by the low AUC; the basis of this pilot study might pave the way to improve the diagnostic performances of this device. The high false-negative rates could certainly limit the electronic nose as a screening tool; further device development and larger sample sizes could reduce this limitation. Another significant limitation of the study was represented by the selection of control patients and the fact that they never underwent cystoscopy to rule out cancer; the controls had no suspicions of bladder cancer (hematuria or symptoms consistent with a bladder lesion) that could justify a complete workup for bladder cancer and even a cystoscopy. We did not repeat the analysis of the samples when voided at different time points from the same patients; it represented a significant limitation but surely might represent a further line of research deserving full consideration. We did not present the data on tandem analysis from the same specimen, and it was one of the most important limitations of the study; surely, the comparison of the results with tandem analysis might represent one of the further lines of research for the optimal development of the device.

It is clear that we are at the beginning, but we think that the electronic nose has several potential advantages: it is quick, it is not dependent on the operator, and it could be useful in cases of inconclusive findings on cystoscopy/cytology/FISH. The measurements might allow the realization of a database of digitized patterns in order to follow up the same patient over time. Surely, an established sensitivity/specificity parameter selection might be universalized in order to make this technique more broadly applicable. Furthermore, the establishment of a sort of correlation with cytology might lead to improved diagnostic accuracy. Looking at the bigger picture, it would be more helpful to follow patients who may have initial results when a tumor is present and different results after it has been resected or treated.

Since NMIBC encompasses a really broad clinical scenario, the exploratory nature of this research deserves to be considered first as an initial step toward the future directions of the development of this tool. One of the possible scenario in which the tool could be mostly useful is in the follow-up of NMIBC patients (including BCG-treated patients): the diagnostic accuracy of the electronic nose might delay the interval between endoscopic controls, and it might confirm (or not) the indication of further exams such as in the case of negative cytology and synchronous positive e-nose results (indication to perform endoscopy) or in the case of negative cytology and synchronous negative e-nose results (indication to not perform endoscopy). As for hematuria, it is still widely regarded as a confounding factor: when the electronic nose reaches an optimal level of standardization, it may play a role in this scenario.

Moreover, the standardization of the method might be relevant in terms of health economics; a new effective and non-invasive diagnostic test could be useful as an additional tool or as a replacement for standard diagnostic procedures, with considerable potential cost savings.

The promising results of the study might make this tool worthy to be prospectively studied in a randomized trial; in this way, it might allow reaching the optimal level of standardization and the test can be considered safe and reliable to be integrated into clinical practice.

## 5. Conclusions

The aim of the study was the clinical evaluation of the diagnostic performance of a VOC sensor device in BCa. Once validated by an updated statistical method and after further necessary refining of the test (according to recent advances in all fields, including statistical techniques and methods for data analysis), electronic nose technology might be used in the future for screening, diagnosis, assessment of the treatment response, or even staging. Once the device is standardized, especially regarding possible confounders, it might be even used in the initial evaluation in the case of gross hematuria.

This pilot study might pave the way for further trials designed to detect the best sensor, and then to select a panel of sensor tests to use for detection of BCa, by narrowing down to those defined as more responsive and appropriate. The development and optimization of a non-invasive, repeatable, accurate, potentially cost-effective method in BCa diagnosis, such as the use of an electronic nose, might improve patient care.

## Figures and Tables

**Table 1:** Baseline characteristics.

	Group 1 (BCa Patients)	Group 2 (Controls)	*p*-Value
No. of patients	102	96	
Age, mean years ± SD (range)	70.8 ± 12.4 (33–93)	68.3 ± 10.7 (42–86)	0.29
Gender, no. of patients (%)			0.56
Men	87 (85.3%)	79 (82.3%)	
Women	15 (14.7%)	17 (17.7%)	
Smoking, no. of patients (%)			
Non-smoker	10 (9.8%)	16 (16.7%)	0.15
Former smoker	45 (44.1%)	31 (32.3%)	0.16
Smoker	47 (46.1%)	49 (51%)	0.49
Comorbidities, no. of patients (%)			
Arterial hypertension	56 (54.9%)	41 (42.7%)	0.08
Diabetes mellitus	22 (21.6%)	16 (16.7%)	0.38
History of AMI	21 (20.6%)	14 (14.6%)	0.27
Kidney failure	9 (8.8%)	4 (4.2%)	0.18
COPD	16 (15.7%)	9 (9.4%)	0.18
Liver disorder	4 (3.9%)	1 (1%)	0.20
Dyslipidemic condition	21 (20.6%)	12 (12.5%)	0.13
Other neoplastic disease	11 (10.8%)	6 (6.2%)	0.25

SD: standard deviation; AMI: acute myocardial infarction; COPD: chronic obstructive pulmonary disease.

**Table 2:** Tumor characteristics.

	Total	NMIBC	MIBC	*p*-Value
Histology, no. of patients (%)				0.00
Pure urothelial	95 (93.1%)	80 (98.8%)	15 (71.4%)	
With differentiation	7 (6.9%)	1 (1.2%)	6 (28.6%)	
Grade, no. of patients (%)				0.00
Low	31 (30.4%)	31 (38.3%)	-	
High	71 (69.6%)	50 (61.7%)	21 (100%)	
Stage, no. of patients (%)				
pTa	36 (35.3%)	36 (44.4%)	-	
pT1	33 (32.3%)	33 (40.7%)	-	
Solitary pCis	9 (8.8%)	9 (11.1%)	-	
Concurrent pCis	3 (2.9%)	3 (3.7%)	-	
pT2	16 (15.7%)	-	16 (76.2%)	
≥pT3	5 (4.9%)	-	5 (23.8%)	
Focality, no. of patients (%)				0.11
Monofocality	43 (42.2%)	31 (38.3%)	12 (57.1%)	
Multifocality	59 (57.8%)	50 (61.7%)	9 (42.9%)	
Mean size, cm ± SD (range)	2.7 ± 1.31 (1–6)	2.4 ± 1.2	3.7 ± 1.3	0.00
First diagnosis, no. of patients (%)	43 (42.2%)	33 (40.7%)	10 (47.6%)	0.12
Median time from 1st diagnosis, year ± SD (range)	1.8 ± 1.9 (0–5)	1.9 ± 2.0 (0–5)	1.2 ± 1.7 (0–5)	0.43

NMIBC: non-muscle invasive bladder cancer; MIBC: muscle invasive bladder cancer; Cis: carcinoma in situ; SD: standard deviation.

**Table 3:** Comparison of accuracy, sensitivity, and specificity.

	Accuracy, %	Sensitivity, %	Specificity, %
GC-MS [8,15,16]	70–100%	70–100%	42–97%
Sniffer dogs [5,6]	70–90.1%	55–86%	56–92%
Electronic nose [19]	86.7–93.3%	93.3%	86.7%
Our experience	78.8% (71.6–87.5)	91.1% (72.5–100)	89.1% (81–95.8)

GC-MS: gas chromatography mass spectrometry.

**Table 4:** Comparison of the e-nose to other urine-based biomarkers regarding sensitivity and specificity.

Test	Target of Measurement/Mechanism of Detection	Sensitivity, % (Range)	Specificity % (Range)
NMP22 BladderChek * [28,29]	Measurement of nuclear matrix proteins (quantitative ELISA)	(11–85.7%)	(77–100%)
NMP22 * [28,29]	Measurement of nuclear matrix proteins (qualitative point-of-care test)	(24–81%)	(49–100%)
BTA STAT * [28,29]	Measurement of human complement factor-H-related protein (point-of-care test)	(40–72%)	(29–96%)
BTA track * [28,29]	Measurement of human complement factor-H-related protein (quantitative ELISA)	(50–62%)	(68–87%)
Immunocyt * [28,29]	Fluorescent test combining 3 monoclonal antibodies (M344, LDQ10, 19A211)	(50–85%)	(62–86%)
UroVysion * [28,29]	Measurement of aneuploidy for chromosomes 3, 7, and 17 and loss of the 9p21 locus via fluorescence in situ hybridization (FISH)	(13–100%)	(63–100%)
Cxbladder monitor [28,29]	Measurement of 5 urine mRNA biomarkers and 2 clinical variables	(91–93%)	-
Bladder cancer (UBC) test [28,29]	Measurement of cytokeratins 8 and 18	(12–80%)	(77.3–97%)
EpiCheck [28,29,30]	DNA methylation (15 biomarkers) changes	86% (excluding Ta-LG)	86%
ADXBLADDER [28,29,31]	Detection of MCM5 antibodies	73.5% (62.7–82.6)	33.3% (18.6–51)
Our experience	VOCs’ detection	91.1% (72.5–100%)	89.1% (81–95.8%)

* FDA-approved urinary assays to use alongside cystoscopy for diagnosis and surveillance.

**Figure A1:**
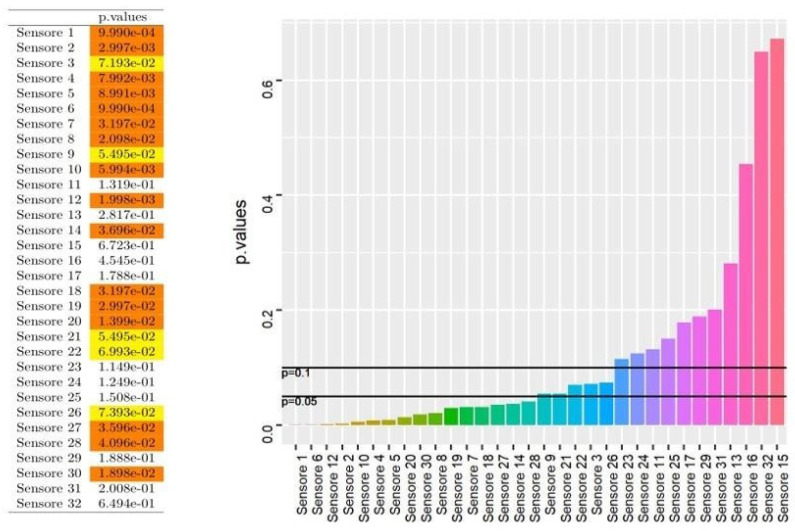
Measurement patterns identified (baseline characteristics).

## Data Availability

The data presented in this study are available on request from the corresponding author.

## Appendix A

We reported statistically significant differences in VOC measurement-identified patterns (by considering all subjects) between controls and BCa patients, both male and female (8/32 and 28/32 sensors, respectively); 28/32 (87.5%) sensors showed significant differences between controls and BCa patients by combining results for both sexes (Figure A1).

We reported statistically significant differences in VOC measurement-identified patterns (by considering all subjects) between controls and BCa patients, both smokers and non-smokers (30/32 and 19/32 sensors, respectively); 30/32 (93.75%) sensors showed significant differences by combining the results for the three smoking categories (non-smokers, formers, and smokers). None of the sensors revealed a potential predictor role of previous exposure to tobacco smoke.

We reported statistically significant differences in VOC measurement-identified patterns (by considering all subjects) between controls and BCa patients, both without and with comorbidities: arterial hypertension (25/32 sensors, 78.1%), diabetes mellitus (27/32 sensors, 84.4%), a history of AMI or kidney failure (in both cases 19/32 sensors, 59.4%), chronic obstructive pulmonary disease (COPD) (27/32 sensors, 84.4%), or other neoplastic diseases (14/32 sensors, 43.8%).

We did not report statistically significant differences by analyzing the presence of liver disorders or dislipidemic conditions due to the small number of controls.

Statistically significant differences were also associated with the presence of diabetes mellitus for 12/32 (37.5%) sensors, a history of AMI for 1/32 (3.1%) sensors, kidney failure for 3/32 (9.4%) sensors, and COPD for 4/32 (12.5%) sensors. We did not report statistically significant differences between patients with monofocal and multifocal tumors (Figure 2d) or in cases of arterial hypertension, liver disorders, or dyslipidemic conditions (mean *p* > 0.05).
